# Photosynthetic Pigments and Chlorophyll Fluorescence of White-Fleshed (*Hylocereus undatus*) and Red-Fleshed (*Hylocereus costaricensis*) Pitaya Following Foliar Application of Different Mineral Formulations Under Semi-Arid Conditions

**DOI:** 10.3390/plants15213495

**Published:** 2026-11-09

**Authors:** Raires Irlenizia da Silva Freire, Agda Malany Forte de Oliveira, Renata Ramayane Torquato Oliveira, David Emanoel Gomes da Silva, Matheus Augusto Silva, Daise Feitoza da Rocha, Joilma Maria de Souza, Elisandra Sampaio de Freitas, Anderson Rodrigo Luciano da Silva, Jéssica Crhistie de Castro Granjeiro Oliveira, Lucas Rodrigues Bezerra da Silva, Adriano do Nascimento Simões, Fred Augusto Lourêdo de Brito, Francisco Vanies da Silva Sá, Vander Mendonça

**Affiliations:** 1 Department of Agricultural and Forestry Sciences, Federal Rural University of the Semi-Arid Region, Mossoró 59625-900, RN, Brazil; rairessilva10@hotmail.com (R.I.d.S.F.); renataramayanet@gmail.com (R.R.T.O.); davidemanoel2110@gmail.com (D.E.G.d.S.); matheus.silva95924@alunos.ufersa.edu.br (M.A.S.); daiserocha11@gmail.com (D.F.d.R.); joilmasouza17@hotmail.com (J.M.d.S.); elisandrasmp@gmail.com (E.S.d.F.); anderson.silva22447@alunos.ufersa.edu.br (A.R.L.d.S.); jessicacrhistie@gmail.com (J.C.d.C.G.O.); lucasrodrigues86dix@gmail.com (L.R.B.d.S.); fred.louredo@ufersa.edu.br (F.A.L.d.B.); vander@ufersa.edu.br (V.M.); 2 Postgraduate Program in Agricultural Sciences, Paraiba State University, Campina Grande 58429-500, PB, Brazil; 3 Academic Unit of Serra Talhada (UAST), Federal Rural University of Pernambuco, Serra Talhada 56909-535, PE, Brazil; adriano.simoes@ufrpe.edu.br; 4 Department of Agrarian and Exact Sciences, Paraíba State University, Catolé do Rocha 58884-000, PB, Brazil; vanies_agronomia@hotmail.com

**Keywords:** photooxidative stress, Bordeaux mixture, calcium silicate, JIP-test, photoprotection

## Abstract

High air temperatures and intense solar radiation in semi-arid regions may impose thermal and photooxidative challenges on cacti. This study evaluated the physiological responses of white-fleshed (*Hylocereus undatus*) and red-fleshed (*Hylocereus costaricensis*) pitaya to foliar application of calcium sources and sunscreens. The experiment used a randomized block design (4 × 2 factorial) and tested four strategies (control, SunGard^®^, calcium silicate, and Bordeaux mixture) on the two pitaya species. In red-fleshed pitaya, Bordeaux mixture was associated with the highest contents of chlorophyll a, chlorophyll b, total chlorophyll, and carotenoids, with increases of approximately 101.3%, 114.4%, 108.3%, and 99.4%, respectively, relative to the control, while reducing energy dissipation (DI_0_/RC = 0.42) and increasing the photosynthetic performance index (PIabs) by 62.0%. In contrast, white pitaya showed a marked increase in the quantum yield of dissipation (φD_0_) and DI_0_/RC (1.53 with Bordeaux mixture), indicating treatment-dependent changes in excitation-energy partitioning. Maximum photochemical efficiency (*Fv*/*Fm*) remained high in both species (approx. 0.81–0.84) under calcium silicate and SunGard^®^ treatments. Foliar mineral formulations modified photosynthetic pigment contents and fluorescence responses in a species-dependent manner under semi-arid field conditions; the observed changes do not, by themselves, establish increased thermotolerance. Overall, the foliar formulations elicited distinct species-dependent responses in pigment accumulation, energy dissipation, and PSII photochemical performance under the recorded semi-arid field conditions.

## 1. Introduction

Pitaya (*Hylocereus* spp.) is a high-value cactus crop whose production has grown rapidly in domestic and international markets due to increasing demand for fruits with high nutritional and functional quality [1]. Its attractive visual and nutritional features, appealing coloration, and pleasant flavor, along with its richness in bioactive compounds such as betalains and flavonoids, drive the crop’s commercial acceptance [2].

In Brazil, cultivation of this species has expanded into various regions, including semi-arid environments, because it adapts to adverse climatic conditions and can diversify agricultural systems [3]. These adaptations include morphophysiological traits such as water storage capacity in the cladodes, the absence of leaves, and, in some species, a thick waxy layer, which support plant development in hot, dry climates and make pitaya a promising alternative for these farming systems [4].

Under open-field cultivation in semi-arid regions, high temperatures and intense solar radiation may challenge the photosynthetic apparatus and favor thermal and photooxidative stress [5,6,7]. Excessive light absorption triggers overproduction of reactive oxygen species (ROS), degrading photosynthetic pigments, damaging the oxygen-evolving complex (OEC), and photoinhibiting photosystem II (PSII) [7]. These physiological changes manifest as yellowing or sunburn (scald) on cladodes and a decrease in maximum quantum yield (*Fv*/*Fm*), compromising photosynthetic performance, plant growth, and fruit quality [8,9,10].

The photosynthetic apparatus responds differently to the combined effects of high temperature and elevated irradiance across species and cultivars, underscoring the need to understand the physiological mechanisms underlying heat tolerance and develop strategies to preserve photosynthetic function under stress [11,12].

To mitigate these adverse effects on the photosynthetic apparatus, exogenous application of calcium (Ca^2+^) and mineral sunscreens has emerged as a promising approach [13]. Calcium plays vital structural and regulatory roles, stabilizing biological membranes and acting as a key secondary messenger in plant stress signaling pathways [14]. In addition to calcium, other nutrients in the applied formulations, such as silicon and sulfur, can also enhance heat stress tolerance by helping preserve the photosynthetic apparatus and membrane integrity [15,16]. Under heat stress, Ca^2+^ influx activates protective signaling cascades that preserve cell integrity, support PSII photochemical efficiency, reduce oxidative damage, and modulate stomatal conductance to regulate tissue surface temperature [17,18].

However, the efficacy of calcium-based strategies depends heavily on the specific source and formulation used. Calcium formulations, including calcium hydroxide, calcium carbonate, and calcium silicate, have been employed to alleviate solar injury, yielding distinct responses across sources. With calcium silicate, the co-supplied silicon further helps maintain PSII photochemical efficiency and mitigate heat-induced oxidative stress [17]. Researchers have also investigated reflective mineral formulations for surface protection in other fruit crops exposed to high solar radiation and temperature, including kiwifruit and orange [19,20,21].

Although researchers have investigated calcium in relation to abiotic stress tolerance, direct comparisons of calcium-containing foliar formulations in the two pitaya species under the same semi-arid field conditions remain limited, particularly when photosynthetic pigments are evaluated alongside OJIP fluorescence kinetics and JIP-test parameters. These formulations also differ in their additional mineral constituents and potential physical surface effects, so researchers cannot attribute their responses exclusively to calcium. Therefore, we asked whether the two pitaya species, white-fleshed (*Hylocereus undatus*) and red-fleshed (*Hylocereus costaricensis*), exhibit different pigment and fluorescence responses to the foliar formulations. We hypothesized that the magnitude and direction of the photosynthetic responses would depend on the interaction between pitaya species and foliar treatment under semi-arid field conditions.

Accordingly, this study aimed to compare photosynthetic pigment contents and chlorophyll fluorescence responses of white- and red-fleshed pitaya species to different calcium-containing foliar formulations under semi-arid field conditions.

## 2. Results

### 2.1. Photosynthetic Pigments

**Figure 1:**
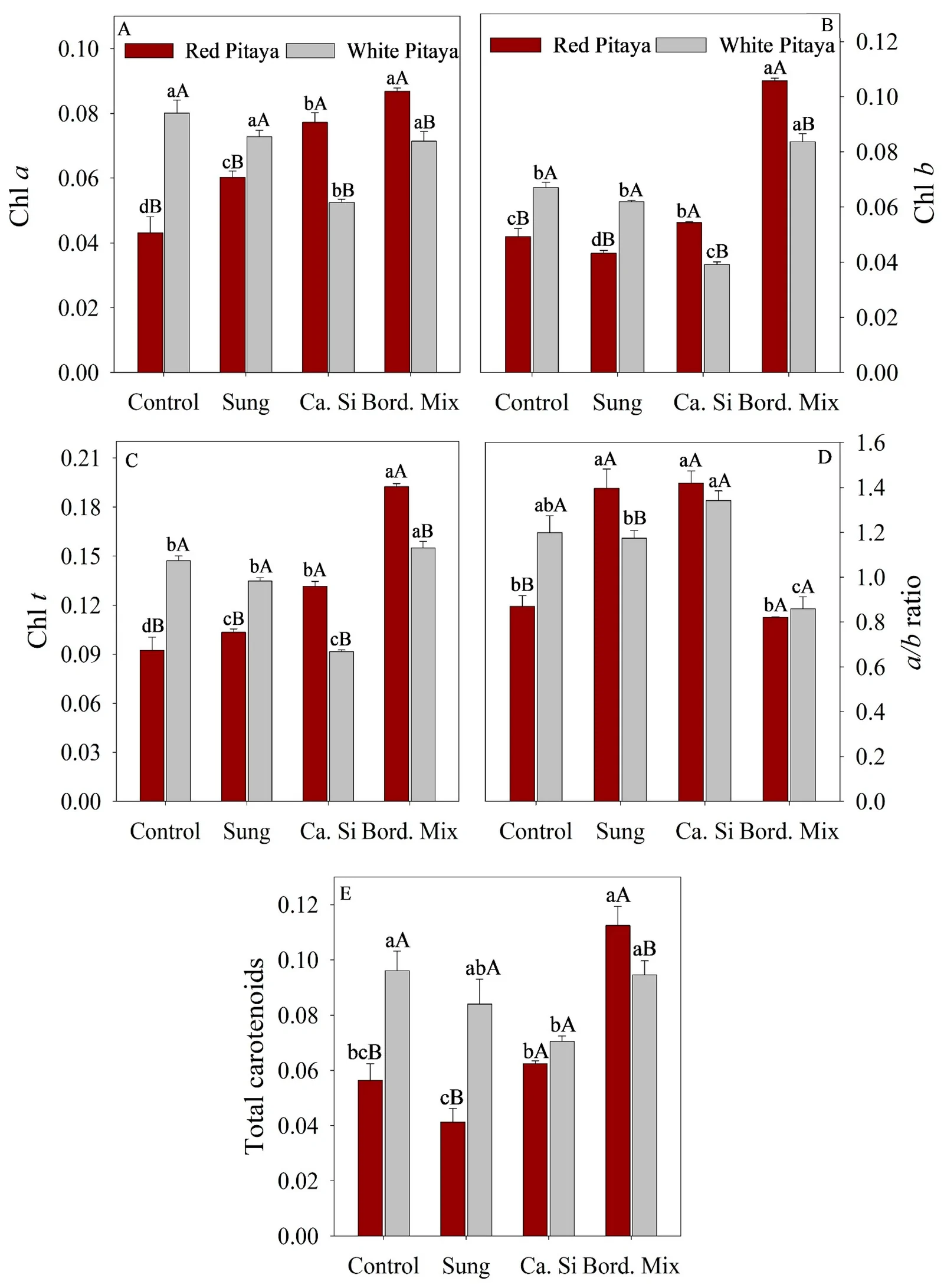
Chlorophyll *a* (**A**), chlorophyll *b* (**B**), total chlorophylls (**C**), chlorophyll *a*/*b* ratio (**D**), and total carotenoid (**E**) contents in cladodes of red-fleshed (*Hylocereus costaricensis*) and white-fleshed (*Hylocereus undatus*) pitaya subjected to foliar application of different calcium sources. Means followed by distinct lowercase letters differ significantly within each species across foliar treatments, and distinct uppercase letters differ significantly between species within each foliar treatment by the LSD test (*p* ≤ 0.05). Vertical bars represent the standard error of the mean (*n* = 4). Sung = SunGard^®^, Ca.Si = calcium silicate and Bord. Mix = Bordeaux mixture.

The interaction between calcium sources and pitaya species significantly influenced photosynthetic pigment contents (*p* ≤ 0.05) (Figure 1). In red-fleshed pitaya (*Hylocereus costaricensis*), the control treatment (no thermal protector) resulted in the lowest Chl *a* (0.043 mg g^−1^ FM), Chl *b* (0.049 mg g^−1^ FM), and Chl t (0.092 mg g^−1^ FM) contents (Figure 1A–C). However, applying calcium sources induced chlorophyll accumulation in this variety, particularly with Bordeaux mixture, which increased Chl *a*, Chl *b*, and Chl *t* contents by 101.3%, 114.4%, and 108.3%, respectively, compared to the control (Figure 1A–C). In white-fleshed pitaya (*H. undatus*), chlorophyll responses differed from those observed in the red-fleshed species. Chlorophyll *a* contents were statistically similar under the control, SunGard^®^, and Bordeaux mixture treatments, whereas calcium silicate resulted in a lower value (0.052 mg g^−1^ FM). Bordeaux mixture produced the highest chlorophyll *b* content (0.084 mg g^−1^ FM), while calcium silicate produced the lowest (0.039 mg g^−1^ FM). Total chlorophyll was also highest under the Bordeaux mixture (0.155 mg g^−1^ FM) and statistically similar to the control. In contrast, calcium silicate resulted in the lowest content (0.092 mg g^−1^ FM) (Figure 1A–C).

The interaction between foliar treatments and pitaya species significantly affected the chlorophyll a/b ratio (Figure 1D). The calcium sources similarly influenced the chlorophyll *a*/*b* ratio in both species (Figure 1D). In red-fleshed pitaya, SunGard^®^ (1.40) and calcium silicate (1.42) produced the highest *a*/*b* ratios, while the control (0.87) and Bordeaux mixture (0.82) produced lower values. Conversely, in white-fleshed pitaya, calcium silicate produced the highest *a*/*b* ratio (1.34), while Bordeaux mixture resulted in the lowest ratio (0.86). Under control conditions, white-fleshed pitaya maintained a higher *a*/*b* ratio than red-fleshed pitaya, whereas red-fleshed pitaya reached a higher ratio under SunGard^®^ application. Under calcium silicate and Bordeaux mixture treatments, the a/b ratio did not differ significantly between species (Figure 1D).

Total carotenoid contents also showed a significant treatment × species interaction (*p* = 0.0001; Figure 1E). In red-fleshed pitaya, the Bordeaux mixture produced the highest carotenoid content (0.112 mg g^−1^ FM), approximately 99.4% higher than the control (0.056 mg g^−1^ FM). SunGard^®^ resulted in the lowest value (0.041 mg g^−1^ FM), whereas calcium silicate (0.063 mg g^−1^ FM) did not differ significantly from the control. In white-fleshed pitaya, the control and Bordeaux mixture treatments showed statistically similar carotenoid contents (0.096 and 0.095 mg g^−1^ FM, respectively). SunGard^®^ and calcium silicate resulted in lower values, with calcium silicate showing the lowest content (0.071 mg g^−1^ FM) (Figure 1E).

### 2.2. Chlorophyll Fluorescence

**Figure 2:**
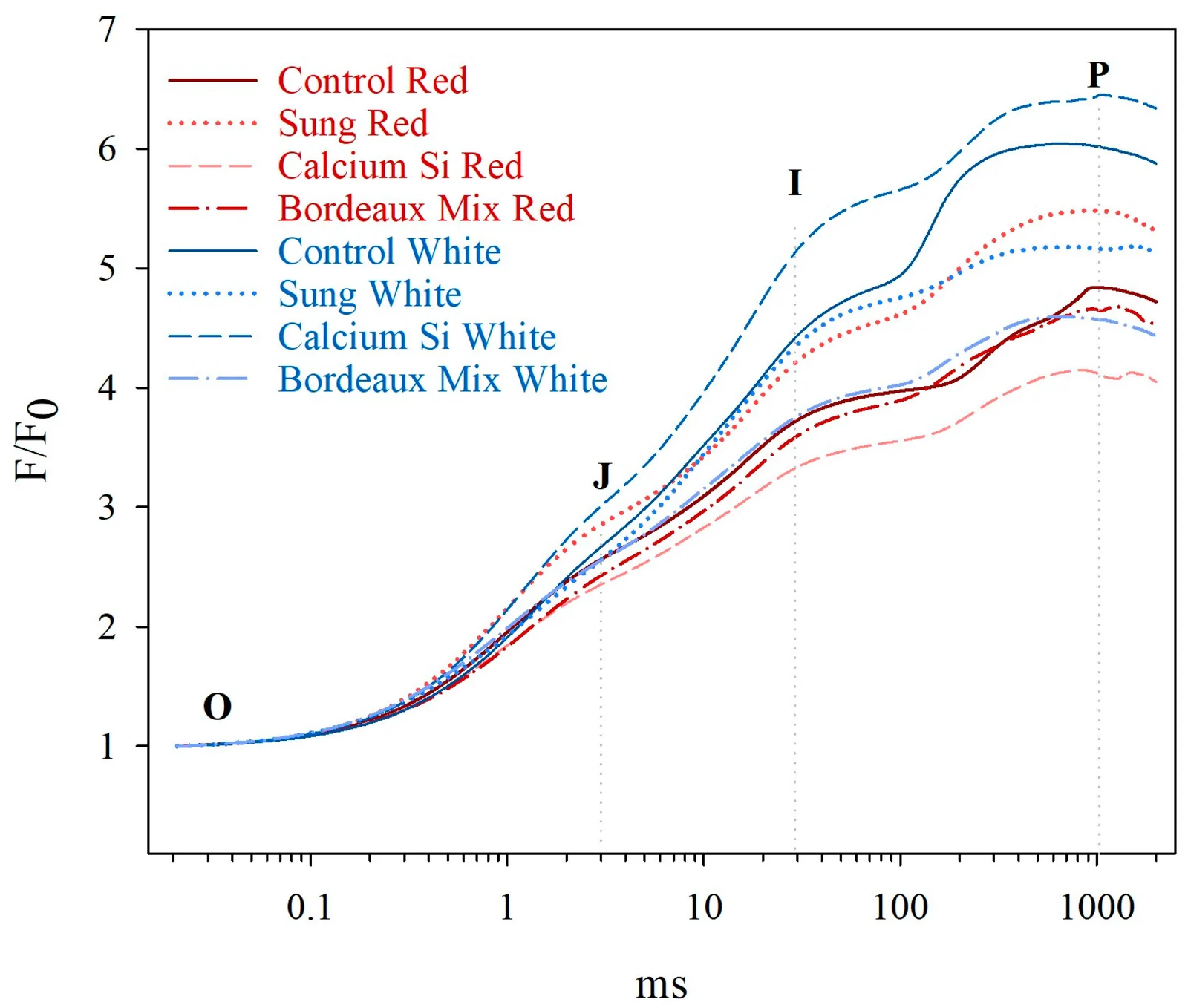
Chlorophyll *a* fluorescence transient induction curves (OJIP curves) in cladodes of red-fleshed (*Hylocereus costaricensis*) and white-fleshed (*Hylocereus undatus*) pitaya subjected to foliar application of different calcium sources. Points O, J, I, and P represent the fluorescence intensity plotted against time (ms) on a logarithmic scale. Sung = SunGard^®^, Ca.Si = calcium silicate and Bord. Mix = Bordeaux mixture.

The transient kinetic curve of chlorophyll *a* fluorescence (OJIP curve) showed a higher overall fluorescence amplitude across all phases (OJIP) in white-fleshed pitaya plants than in red-fleshed pitaya (Figure 2). Differences among treatments began in the J and I phases, with the greatest divergence at the P phase (maximum fluorescence). At this phase, calcium silicate and SunGard^®^ treatments yielded the highest F/F_0_ values for white- and red-fleshed pitaya, respectively (Figure 2).

### 2.3. Biophysical Parameters of the JIP-Test and Photochemical Efficiency

**Figure 3:**
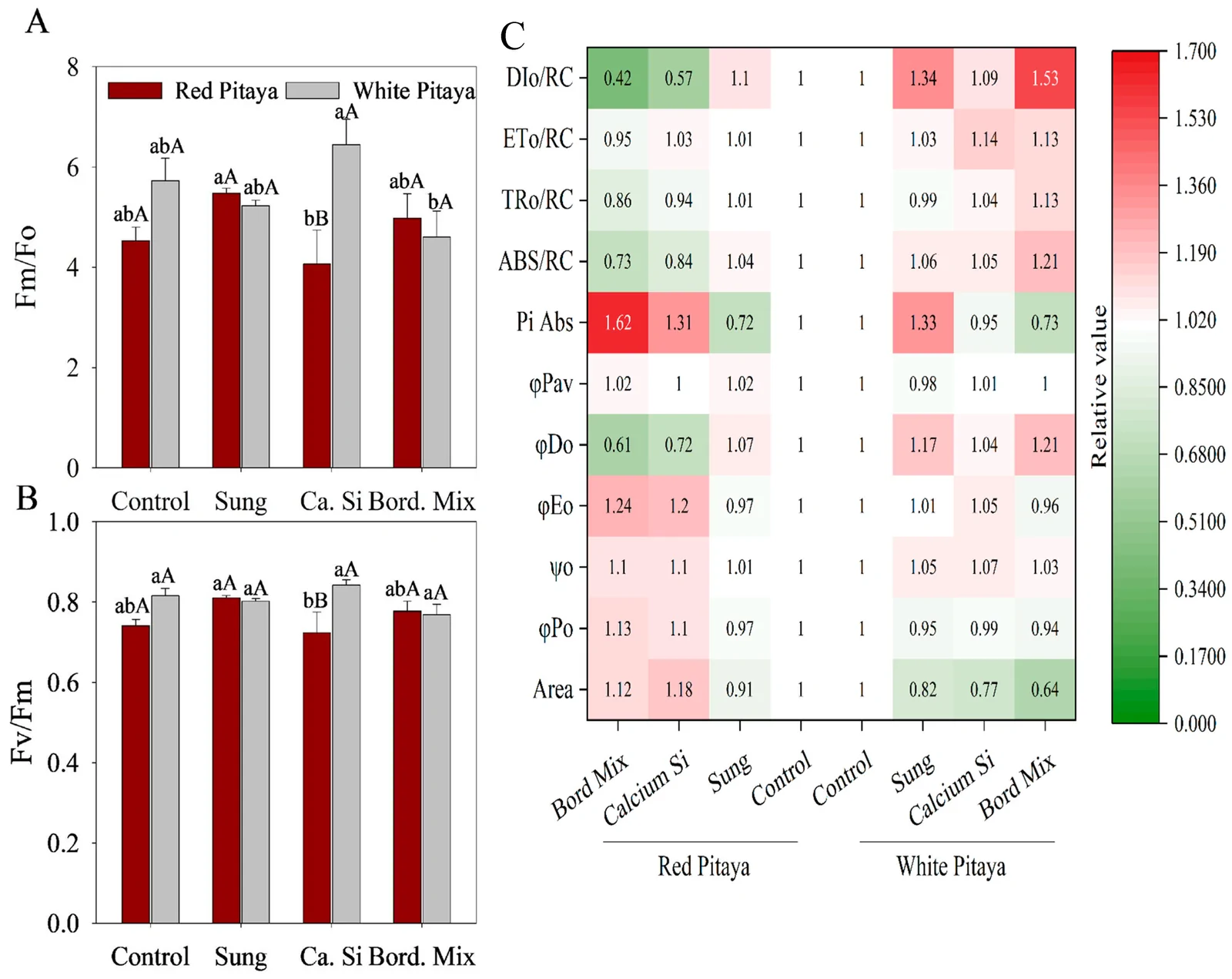
Maximum-to-minimum fluorescence ratio (*Fm*/*F*_0_) (**A**), maximum photochemical efficiency of photosystem II (*Fv*/*Fm*) (**B**), and JIP-test parameters expressed as ratios normalized to the respective species-specific control (=1.0) presented as a heatmap (**C**) in cladodes of red-fleshed (*Hylocereus costaricensis*) and white-fleshed (*Hylocereus undatus*) pitaya subjected to foliar application of different calcium sources. Means followed by distinct lowercase letters differ significantly within each species across foliar treatments, and distinct uppercase letters differ significantly between species within each foliar treatment by the LSD test (*p* ≤ 0.05). Sung = SunGard^®^, Ca.Si = calcium silicate and Bord. Mix = Bordeaux mixture.

The *Fm*/*Fo* ratio varied substantially between species and calcium sources (Figure 3A). White-fleshed pitaya showed the highest values with calcium silicate application, reaching approximately 6.5, whereas the red-fleshed variety reached about 4.1 under the same treatment. In contrast, SunGard^®^ application produced the highest *Fm*/*Fo* values in red-fleshed pitaya, reaching approximately 5.5. These results suggest differences among the studied species in how the photosynthetic apparatus responds and how they use absorbed light energy under high-temperature conditions.

Maximum quantum efficiency values of photosystem II (*Fv*/*Fm*) ranged from 0.72 to 0.84 across all treatments (Figure 3B). White-fleshed pitaya showed the highest values under calcium silicate (≈0.84), whereas the red-fleshed variety performed best with SunGard^®^ application (≈0.81) (Figure 3B).

The heatmap normalized the JIP-test parameters to the respective species-specific control and assigned a relative value of 1.0 (Figure 3C). Therefore, values above or below 1.0 indicate relative increases or decreases, respectively, rather than absolute values of the physiological parameters. In red-fleshed pitaya (*Hylocereus costaricensis*), the Bordeaux mixture was associated with a relative DI_0_/RC value of 0.42 (58% decrease) and a relative PIabs value of 1.62 (62% increase). In contrast, white-fleshed pitaya (*Hylocereus undatus*) showed a relative DI_0_/RC value of 1.53 and a relative φD_0_ value of 1.21 under Bordeaux mixture, representing increases of 53% and 21%, respectively, compared with the species-specific control. These changes were accompanied by lower relative PIabs (0.73) and OJIP curve area (0.64), corresponding to decreases of 27% and 36%, respectively. Thus, the heatmap indicates contrasting patterns of energy dissipation and relative photochemical performance between the two species under the evaluated treatments.

## 3. Discussion

Pitaya’s photosynthetic responses to foliar mineral formulations are particularly relevant in semi-arid environments with high daytime temperatures and substantial solar radiation [5,7,22]. In the present study, treatment effects on pigment composition and chlorophyll a fluorescence varied between species. Following Bordeaux mixture application, red-fleshed pitaya (*H. costaricensis*) exhibited higher relative PIabs and lower relative DI_0_/RC. In contrast, white-fleshed pitaya (*H. undatus*) showed increased relative energy dissipation accompanied by a reduction in relative PIabs (Figure 3C). These responses indicate contrasting patterns of photochemical energy utilization and dissipation associated with the interaction between species and foliar formulations.

Meteorological records further characterize the environmental conditions preceding and coinciding with the physiological assessment on 10 November 2025. Between 1 and 10 November, daily maximum air temperatures averaged 34.53 °C (range: 32.64–36.64 °C), daily global solar radiation averaged 21.26 MJ m^−2^ day^−1^, and no precipitation was recorded. On the assessment date, mean, maximum, and minimum air temperatures were 28.09, 34.26, and 23.33 °C, respectively, with mean relative humidity of 58.44%, daily global solar radiation of 21.09 MJ m^−2^, and sunshine duration of 11.43 h. These environmental descriptors provide a quantitative basis for interpreting the species-dependent variation in photosynthetic pigment status and PSII fluorescence under semi-arid field conditions.

Although pitaya is adapted to warm environments, it generally grows best at 20–30 °C, and maximum net CO_2_ assimilation in *H. undatus* has been reported under a day/night temperature regime of 30/20 °C [23,24]. Prolonged exposure to 35/25 °C can reduce carbon assimilation, whereas more severe thermal regimes (40/30 °C and 45/35 °C) have been associated with physiological impairment, cladode damage, and reduced photosystem II photochemical efficiency in *Hylocereus* spp. [8,23,25]. In this context, the elevated air temperatures recorded during the experimental period, particularly in early November, when daily maxima frequently exceeded 30 °C, combined with substantial solar radiation, indicate environmental conditions potentially conducive to thermal and photooxidative stress. These conditions provide a physiological context for understanding the two pitaya species’ differential responses to the foliar formulations evaluated in this study.

In untreated red-fleshed pitaya, chlorophyll and carotenoid contents differed from those observed after foliar application of the mineral formulations (Figure 1). High irradiance and temperature can influence pigment metabolism, thylakoid organization, and photosynthetic electron transport [26,27], while reactive oxygen species may contribute to changes in photosynthetic complexes [28,29]. The contrasting pigment responses may reflect differences in species-specific physiology and in the composition and physical properties of the applied formulations. Bordeaux mixture contains copper sulfate and hydrated lime, whereas calcium silicate supplies both calcium and silicon, and SunGard^®^ is a commercial mineral formulation containing calcium and magnesium hydroxides and additives. These treatments therefore cannot be interpreted as isolated calcium effects.

Calcium contributes to the structural stability of biological membranes and cell walls by forming pectate cross-links. It acts as a secondary messenger in cellular signaling pathways involved in heat-stress acclimation [30,31,32]. Silicon helps preserve thylakoid integrity and attenuates heat-induced oxidative damage [15,33]. Additionally, Bordeaux mixture supplies copper, an essential component of plastocyanin, which mediates electron transfer from the cytochrome b_6_f complex to photosystem I (PSI) in the thylakoid lumen, thereby supporting linear electron transport and helping protect the photosynthetic apparatus against photo-oxidative damage [34,35]. Furthermore, sulfate contributes to the synthesis of sulfur-containing amino acids and glutathione, strengthening cellular antioxidant capacity under photo-oxidative stress [36,37].

Beyond the specific effects of individual mineral elements, the pronounced accumulation of photosynthetic pigments in red-fleshed pitaya following Bordeaux mixture application may be associated with the combined presence of Cu, S, and Ca in the formulation. Copper is an essential component of plastocyanin, which mediates electron transfer from the cytochrome b_6_f complex to photosystem I [34,35]. Sulfur is involved in the biosynthesis of cysteine, methionine, and glutathione, which contribute to plant antioxidant metabolism and reactive oxygen species (ROS) scavenging [36,37]. However, because nutrient uptake, plastocyanin content, and antioxidant activity were not directly assessed, these mechanisms remain plausible explanations rather than demonstrated causes of the observed responses. Therefore, the present findings do not establish a synergistic biochemical effect among Cu, S, and Ca. Conversely, the higher chlorophyll levels in untreated white-fleshed pitaya indicate distinct pigment-regulation strategies between the two species, with red-fleshed pitaya showing greater metabolic responsiveness to external mineral elicitation to maintain pigment homeostasis under semi-arid conditions.

In white-fleshed pitaya, unlike in red-fleshed pitaya, Bordeaux mixture application maintained chlorophyll *a* content. It promoted a marked increase in chlorophyll *b*, resulting in the highest total chlorophyll content among treatments in this variety (Figure 1B,C). Chlorophyll *b* retention and accumulation play a fundamental role in the stability and organization of photosystem II’s light-harvesting antenna complexes (LHCII) [38]. The chlorophyll *a*/*b* ratio varied according to treatment and species (Figure 1D). In white-fleshed pitaya, the lower chlorophyll *a*/*b* ratio under the Bordeaux mixture reflects greater relative preservation of chlorophyll *b*, which may help organize the antenna complex under field conditions [27,38,39]. Conversely, higher chlorophyll *a*/*b* ratios in SunGard^®^- and calcium silicate-treated red-fleshed pitaya, as well as in calcium silicate-treated white-fleshed pitaya (Figure 1D), indicate a relative reduction in light-harvesting antenna size. This shift in antenna proportion suggests a potential physiological mechanism to limit excess photon absorption under conditions of elevated temperature and solar radiation [27].

Carotenoid contents also showed genotype-dependent dynamics, with a 99.4% accumulation in red-fleshed pitaya treated with Bordeaux mixture relative to the control (Figure 1E). Carotenoids play a key role in photoprotection by quenching triplet chlorophyll states (^3^Chl*), scavenging singlet oxygen (^1^O_2_), and dissipating excess energy as heat via the xanthophyll cycle [24]. As the photosynthetic response of *Hylocereus* spp. to elevated temperatures differs substantially among genotypes [22,25], the carotenoid increase in red-fleshed pitaya treated with Bordeaux mixture was accompanied by a higher relative photosynthetic performance index (PIabs) and lower relative energy dissipation per reaction center (DI_0_/RC), without establishing direct thermal protection (Figure 3C). The higher carotenoid content observed in red-fleshed pitaya under Bordeaux mixture is therefore consistent with a potential change in photoprotective pigment allocation [24]. However, because we did not measure xanthophyll-cycle activity and oxidative markers, the results do not establish enhanced antioxidant protection or greater thermal tolerance. Similarly, the lack of a significant difference between the control and Bordeaux mixture in white-fleshed pitaya does not, by itself, demonstrate more efficient constitutive photoprotection.

Transient chlorophyll *a* fluorescence kinetics (OJIP curve) is a sensitive, non-destructive biophysical tool for monitoring the structural integrity and function of the photosynthetic apparatus under heat stress [40,41,42]. The induction curve consists of sequential phases (O, J, I, and P) that reflect the redox states and electron-transfer dynamics between photosystem II (PSII) and photosystem I (PSI) [42]. In the present study, fluorescence amplitude changes became evident starting in the J (3 ms) and I (30 ms) phases (Figure 2). The fluorescence increase at these intermediate points indicates accumulation of the reduced form of the primary electron acceptor, quinone A (QA-), due to restricted electron transport to secondary acceptors (QB-) on the reducing side of PSII [40].

The maintained photochemical activity in pitaya treated with Bordeaux mixture highlights the synergistic action of the solution’s elements on the electron transport chain. Copper (Cu) plays a structural role in the thylakoid lumen as a cofactor of plastocyanin, the protein that mediates electron transfer from the cytochrome b_6_f complex to the P_700_ reaction center of PSI, ensuring the continuity of linear flow and preventing photo-oxidative overload in PSII [34,43]. Simultaneously, sulfur (S) supplied as sulfate supports photosynthetic metabolism by serving as a precursor for sulfur-containing amino acids (cysteine and methionine) and reduced glutathione (GSH), strengthening reactive oxygen species (ROS) scavenging mechanisms induced by high temperatures [36,37]. This action sustains photochemical functionality without necessarily requiring drastic increases in maximum quantum efficiency (*Fv*/*Fm*).

However, the magnitude of this response may depend on each variety’s physiological characteristics and its capacity to absorb and use the supplied nutrients. In this context, the differential *Fv*/*Fm* response suggests that the species may exhibit distinct photoprotective and antioxidant defense strategies. Red pitaya may rely more on enzymatic antioxidant mechanisms involving the coordinated activity of superoxide dismutase and catalase. In contrast, white pitaya may rely more on flavonoid-mediated photoprotective mechanisms [22].

The divergence observed at the P phase (300 ms to 1 s), corresponding to maximum fluorescence (*Fm*), revealed the pronounced influence of both the mineral source and the genetic material on energy utilization capacity (Figure 2). In white-fleshed pitaya (*H. undatus*), the greater overall curve amplitude and the sharp peak at the P phase under calcium silicate and SunGard^®^ application indicate a higher total reduction capacity of the plastoquinone pool on the acceptor side of PSII. Silicon helps preserve thylakoids and stabilize reaction center proteins [15], while calcium maintains homeostasis and couples the oxygen-evolving complex (OEC) [42,44]. This differentiated response confirms that the photosynthetic apparatus of both *Hylocereus* species processes and partitions excitation energy through distinct biophysical pathways under semi-arid climatic conditions.

White pitaya recorded the highest *Fv*/*Fm* values under calcium silicate application, and red pitaya under SunGard^®^ (Figure 3B), indicating comparatively high maximum photochemical efficiency under the recorded semi-arid field conditions. Although maintaining *Fv*/*Fm* at physiologically adequate levels reflects preserved primary maximum photochemical capacity, integrated analysis of other biophysical parameters reveals shifts in absorbed energy partitioning. The efficacy of exogenous calcium in mitigating high-temperature impacts on PSII corroborates findings in heat-stressed plants [44], where calcium supply preserved PSII functionality, prevented oxygen-evolving complex (OEC) uncoupling, and optimized photosynthetic CO_2_ assimilation. Ca^2+^ stabilizes core PSII proteins and helps regulate cellular signaling cascades that induce heat shock protein (HSP) expression [39,45,46].

In red-fleshed pitaya (*H. costaricensis*), foliar application of Bordeaux mixture and calcium silicate promoted a favorable readjustment of the photosynthetic apparatus, characterized by a reduction in non-photochemical dissipation per reaction center (DI_0_/RC) and simultaneous increases in the photosynthetic performance index (PIabs), quantum yield of electron transport (*φEo*), and efficiency of trapped energy conversion into electron flow (*ψo*) (Figure 3C). The decrease in DI_0_/RC reflects reduced routing of absorbed excitation energy toward non-regulated heat dissipation pathways in active reaction centers. Meanwhile, increases in *φEo* and *ψo* indicate a higher probability and relative efficiency of electron transfer from reduced primary quinone (Q_A_^-^) toward the plastoquinone pool and secondary acceptors (Q_B_^−^) in the transport chain [42,47]. This optimized electron flow increased PIabs, demonstrating an enhanced capacity to convert light energy into chemical energy in red pitaya photosynthesis [42].

The literature reports a favorable effect of calcium silicate on the photosynthetic apparatus, where calcium silicate is recognized for maintaining PSII functionality and electron transport under stress [16]. When evaluating different wheat cultivars under heat stress, [15] observed that response intensity to silicon varied among genetic materials. The species-dependent variation observed in the present study is consistent with these reports and reinforces the importance of considering genetic background when evaluating calcium- and silicon-containing formulations.

In contrast, white-fleshed pitaya routed more energy toward dissipation, mainly under the Bordeaux mixture, as shown by relative increases in DI_0_/RC and φDo, along with reductions in PIabs and OJIP curve area (Figure 3C). The increase in DI_0_/RC may reflect greater energy dissipation in each reaction center, representing a photoprotective response aimed at preventing overload of the photosynthetic apparatus [48,49]. However, the simultaneous reduction in PIabs indicates that this dissipation strategy was associated with lower overall photochemical performance. Thus, increased dissipation should not be interpreted in isolation as PSII damage, but as a shift in energy partitioning that, under the evaluated conditions, was accompanied by reduced photochemical energy conservation efficiency [42,47]. In this case, the concurrent reductions in PIabs and curve area indicate that the higher relative dissipation was accompanied by lower relative photochemical performance.

The higher energy dissipation observed in white pitaya, especially with Bordeaux mixture application, may reflect differences in physiological responses among genetic materials. Recent studies in the genus Hylocereus show that red- and white-fleshed species respond mechanistically differently to environmental stress, with the red variety associated with greater photochemical acclimation capacity under elevated temperatures and irradiance [22]. In this context, the contrasting responses observed in the present study reflect intrinsic differences in how each species regulates and uses absorbed energy through its photosynthetic apparatus.

Taken together, the pigment and fluorescence results show that the mineral formulations altered photosynthetic performance differently in the two pitaya species. In red-fleshed pitaya, lower relative DI_0_/RC, together with higher relative PIabs, φE_0_, and ψ_0_ under Bordeaux mixture and calcium silicate, was consistent with greater allocation of absorbed energy to photochemical processes (Figure 3C). Conversely, white-fleshed pitaya exhibited higher relative DI_0_/RC and φD_0_ under some treatments, particularly Bordeaux mixture, together with lower relative PIabs and OJIP curve area. Consequently, the results suggest that the photosynthetic apparatus responds differently to calcium sources: red pitaya appears more responsive to treatments that favor photochemical energy utilization, whereas white pitaya exhibited greater dissipation under some treatments, sometimes accompanied by reduced photochemical performance.

The present findings characterize species-specific photosynthetic responses during one production cycle under semi-arid orchard conditions. Further studies incorporating temporal monitoring, biochemical indicators of oxidative balance, and reproductive traits, including fruit quality and yield, may help determine the persistence and agronomic relevance of the observed responses. Complementary analyses of antioxidant metabolism and gene expression would also help explain the physiological processes associated with applying these mineral formulations.

## 4. Materials and Methods

### 4.1. Experimental Site Characterization

The experiment was conducted in the experimental orchard of the Department of Fruit Crops at the Federal Rural University of the Semi-Arid (UFERSA), located in Mossoró, Rio Grande do Norte, Brazil (5°11′15″ S, 37°20′39″ W; 18 m altitude), on flat terrain. According to Köppen’s climate classification, the local climate is BSwh (hot semi-arid), with an average annual rainfall of 673.9 mm and an average annual temperature of 27.4 °C [50]. Climatic seasonality is well defined, with a predominant dry season and a short, irregular rainy season.

**Figure 4:**
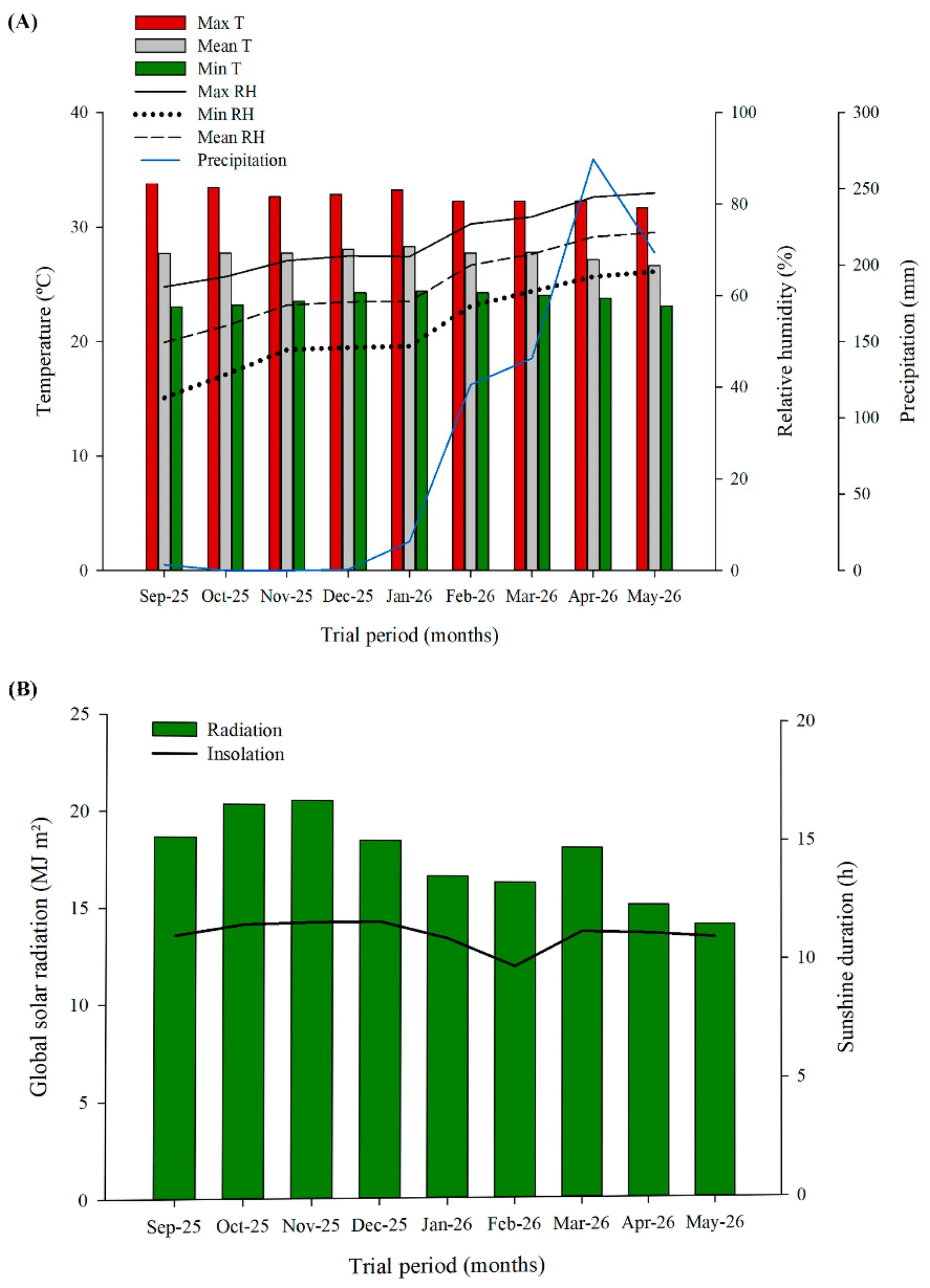
Monthly meteorological conditions during the experimental period (September 2025 to May 2026): (**A**) maximum, mean, and minimum air temperature (°C); maximum, mean, and minimum relative humidity (%); and accumulated precipitation (mm). (**B**) Global solar radiation (MJ m^−2^) and sunshine duration (h).

Meteorological conditions were characterized throughout the experimental period (September 2025 to May 2026) using records of air temperature, relative humidity, precipitation, global solar radiation, and sunshine duration from the available meteorological series for the study site (Figure 4). Additional daily records were analyzed for the physiological assessment performed on 10 November 2025. During 1–10 November, daily maximum air temperature averaged 34.53 °C (range: 32.64–36.64 °C), mean daily global solar radiation was 21.26 MJ m^−2^ day^−1^, and accumulated rainfall was 0 mm. On 10 November, mean, maximum, and minimum air temperatures were 28.09, 34.26, and 23.33 °C, respectively; mean relative humidity was 58.44% (minimum: 35.48%; maximum: 81.40%). Daily global solar radiation totaled 21.09 MJ m^−2^, sunshine duration was 11.43 h, and precipitation was 0 mm.

### 4.2. Plant Material and Cultivation Conditions

The study was carried out throughout the crop production cycle, from September 2025 to May 2026, in the experimental orchard at UFERSA. We evaluated white-fleshed pitaya (*Hylocereus undatus*) and red-fleshed pitaya (*Hylocereus costaricensis*) plants established in the field in March 2018 and propagated by stem cuttings. Plants were spaced at 3.0 m × 2.0 m and supported by wooden posts installed perpendicularly in the soil to 1.8 m, with a wooden cross-arm at the top to support the cladodes in a “T” trellis system. During the training phase, lateral deshooting was performed to maintain a single main stem until reaching the support frame. Before the experimental period, formative pruning stimulated the emergence of new productive shoots.

Fertilization combined cattle manure and mineral fertilizers. Organic manure was applied at 20 kg per plant, split into two equal applications: 10 kg at pruning and 10 kg post-pruning. Mineral fertilization (nitrogen, phosphorus, and potassium) was supplied at 350 g of NPK 16-16-16 per plant, distributed as follows: 150 g after pruning, 100 g at flowering, and 100 g after anthesis. Micro-sprinkler irrigation was supplied via a single emitter per plant, delivering 75 L h^−1^ 60 cm stake (Rondo, Rivulis Irrigation, Kibutz Gvat, Israel) and applying a water depth equivalent to approximately 15 L per plant per week.

### 4.3. Experimental Design and Treatments

The experimental design used was a randomized complete block design in a 4 × 2 factorial arrangement. The first factor consisted of four foliar mineral protection strategies: control (spraying with distilled water only); SunGard^®^ (an agricultural mineral sunscreen composed of 97% micronized calcium and magnesium hydroxide and 3% additives, formulated as a wettable powder); calcium silicate (CaSiO_3_, an inorganic mineral source providing calcium and silicon); and Bordeaux mixture (a protective formulation based on copper sulfate [CuSO_4_] and hydrated lime [Ca(OH)_2_]). The second factor included two pitaya species, *Hylocereus undatus* (white-fleshed) and *Hylocereus costaricensis* (red-fleshed), with four blocks and two plants per plot.

### 4.4. Treatment Application

The spray solutions were prepared immediately before application and applied to the cladodes until runoff, using a 12 L manual knapsack sprayer (XP12, Jacto, Pompeia, Brazil). SunGard^®^ was applied by diluting 120 g of the commercial product in 12 L of water to a concentration of 10 g L^−1^ (1%). The calcium silicate was prepared as a 1.0% (*w*/*v*) suspension by dispersing 120 g of the compound in 12 L of water (10 g L^−1^); because it has low water solubility, the suspension was kept under continuous agitation during application. Bordeaux mixture was prepared at 1% concentration using 120 g copper sulfate and 120 g hydrated lime in 12 L solution. The copper sulfate was dissolved in 5 L of warm water and left to rest. Separately, the hydrated lime was diluted in 2 L of water. The copper sulfate solution was then added to the lime suspension, and the final volume was brought to 12 L. Treatments were applied four times at 15-day intervals, beginning immediately after formative pruning and continuing until the onset of fruit filling. Applications were carried out after 5:00 PM to minimize rapid evaporation and prevent phytotoxicity caused by direct solar radiation.

### 4.5. Variables Analyzed

#### 4.5.1. Photosynthetic Pigments

Photosynthetic pigment content was determined according to the method described by Lichtenthaler [51]. Fresh tissue samples (0.5 g) were macerated and homogenized in 5 mL of 80% (*v*/*v*) acetone. The material was then centrifuged at 6000× *g* for 15 min at 4 °C, and the supernatant was used for spectrophotometric readings. We measured absorbance at 663, 645, and 470 nm using a spectrophotometer (Libra S8, Biochrom, Cambridge, UK). The concentrations of chlorophyll *a* (Chl *a*), chlorophyll *b* (Chl *b*), total chlorophylls (Chl *t)*, and total carotenoids (Car) in the extract (µg mL^−1^) were calculated using Equations (1)–(4). Pigment contents were then converted to a fresh-mass basis (mg g^−1^ FM).

*Chl a* = (12.25 × *A*663) − (2.79 × *A*645) (1)

*Chl b* = (21.50 × *A*645) − (5.10 × *A*663) (2)

*Chl t* = *Chl a* + *Chl b* (3)

(4) $$\mathrm{CAR}=\frac{\left(1000\times A470-1.82\times \mathrm{Chl}\;a-85.02\times \mathrm{Chl}\;b\right)}{198}.$$

#### 4.5.2. Chlorophyll *a* Fluorescence and OJIP Kinetics

Chlorophyll *a* fluorescence measurements and transient induction curves (OJIPs) were measured between 08:00 and 10:00 h on 10 November 2025. During this sampling period, air temperature averaged 28.5 °C as solar irradiance increased. We selected this morning assessment window to record active PSII photochemical behavior and excitation-energy partitioning before extreme midday temperatures (peaking at 34.26 °C later in the afternoon) and associated stomatal closure set in. We evaluated two plants per plot one week after the last treatment application. Measurements were taken using a portable fluorometer, FluorPen FP 100 (Photon Systems Instruments—PSI, Drásov, Czech Republic).

We took readings after a 30 min dark-adaptation period on the cladode surface using dark-adaptation leaf clips to ensure complete oxidation/opening of photosystem II (PSII) reaction centers. We calculated and interpreted biophysical parameters from the transient induction curve (O, J, I, and P phases) using the JIP test, following Strasser [40], as described in Table 1.

### 4.6. Statistical Analysis

The obtained data were subjected to tests for normality [52] and homogeneity using Levene’s test (*p* < 0.05). Once the statistical assumptions were met, the data were subjected to an analysis of variance (ANOVA) with two factors (4 × 2 factorial scheme), including the block effect in a randomized block design (*p* ≤ 0.05). In cases of statistical significance, treatment means were compared using the LSD test (*p* ≤ 0.05). For the JIP test, we normalized fluorescence parameter data relative to the control treatment (control = 1.0) and presented them in a heatmap to facilitate integrated interpretation of energy fluxes. All statistical analyses were performed using SISVAR^®^ software, version 5.6 [53], and graphs were generated using SigmaPlot software (version 15.0).

## 5. Conclusions

Foliar application of mineral formulations induced species-dependent changes in photosynthetic pigments and chlorophyll fluorescence in pitaya cultivated under the recorded semi-arid conditions. In red-fleshed pitaya (*Hylocereus costaricensis*), Bordeaux mixture (1%) was associated with higher pigment contents and a higher relative photosynthetic performance index than the untreated control. In white-fleshed pitaya (*Hylocereus undatus*), certain formulations increased relative energy dissipation, sometimes accompanied by lower relative photochemical performance. These findings show contrasting responses in photosynthetic energy utilization and dissipation and highlight the need to evaluate foliar mineral formulations species, specifically in semi-arid production systems.

## Figures and Tables

**Table 1:** Description of chlorophyll *a* fluorescence and JIP-test parameters used to evaluate the photosynthetic performance of red-fleshed (*Hylocereus costaricensis*) and white-fleshed (*Hylocereus undatus*) pitaya plants.

Parameter	Description
Basic fluorescence parameters	
Area	Area above the fluorescence curve
F_0_	Initial fluorescence
Fm	Maximum fluorescence
Fv	Variable fluorescence
Fm/Fo	Ratio of maximum to minimum fluorescence
Specific energy fluxes per reaction center	
ABS/RC	Energy flux absorbed per reaction center
TR_0_/RC	Energy flux trapped per reaction center
ET_0_/RC	Electron transport flux per reaction center
DI_0_/RC	Energy flux dissipated per reaction center
Quantum yields and efficiencies	
φP_0_	Maximum quantum yield of primary photochemistry
Ψo	Probability of electron transport beyond the primary quinone acceptor
φE_0_	Quantum yield of electron transport
φDo	Quantum yield of energy dissipation
φPav	Average quantum yield of photosynthesis
Performance index	
PIABS	Absorption-based performance index

## Data Availability

The datasets generated and/or analyzed during the current study are available from the corresponding author upon reasonable request.
